# Facial growth and development trajectories based on three-dimensional images: geometric morphometrics with a deformation perspective

**DOI:** 10.1098/rsos.231438

**Published:** 2024-01-10

**Authors:** Yu Jie Zhong, Dan Cui, Patrick Yi Feng Wen, Hai Ming Wong

**Affiliations:** ^1^ Faculty of Dentistry, The University of Hong Kong, 2/F, Prince Philip Dental Hospital, 34 Hospital Road, Sai Ying Pun 7B47, Hong Kong; ^2^ School of Mechanical Engineering, Xi'an Jiaotong University, 28 Xianning W Rd, Xi'an, People's Republic of China; ^3^ School of Life Sciences, Westlake University, 600 Dunyu Road, Hangzhou E10-326, People's Republic of China

**Keywords:** face, growth, Europeans, geometric morphometrics, Procrustes variance, deformation

## Abstract

Developmental changes of facial shape are commonly investigated through geometric morphometrics. A limitation with this approach is the inability to investigate patterns of morphological changes at local scale. This could be addressed through quantifying the deformation required to deform one shape to another. This study aimed to investigate changes in mean, rate and variance of facial shape at local scale using geometric morphometrics through deformation perspective. A total of 2112 Europeans 3 to 40 years old from the three-dimensional Facial Norms project were included. Shape and rate trajectories from partial least-squares regressions revealed that the developmentally protrusive nasal bridge was due to local expansion in surrounding tissues as opposed to shape changes in nasal bridge *per se*. Local expansion of the supraorbital region, in particular the medial part in males, resulted in the sloping forehead and deep-situated eyes with development. Facial shape variation increased nonlinearly with age (*p* < 0.05), with features having larger rate of change becoming more developmentally diversified. In summary, our deformation perspective facilitates unravelling morphogenetic processes underlying shape changes. Our extended analytical scope inspires novel measures worthy of consideration while establishing facial growth charts. The analytical framework in this study is broadly applicable for analysis of shape changes in general.

## 1. Introduction

Understanding facial growth and development attracts interests from a diverse range of disciplines such as medicine, forensics, palaeoanthropology and developmental biology. Facial growth is traditionally studied through changes of a selective set of distances, angles and proportions [1]. Geometric morphometrics is a revolutionary alternative where all landmarks are considered jointly for shape analysis [2]. The method is well suited to investigating facial growth and development [3,4].

Facial shape development, and comparisons of shape differences in general, is usually interpreted and visualized through displacement between corresponding landmarks. Since shape is treated as a property encoded in the entire constellation of landmarks in geometric morphometrics, displacement of individual landmark needs to be interpreted relative to the movement of all other landmarks. Individual landmark displacement is therefore not an independent quantitative trait. Consequently, local shape difference is not appropriately quantified through separate analysis of individual landmarks [5]. Intriguingly, quantification of shape changes locally is essential in unravelling the local morphological effect of the dynamic morphogenetic processes that ultimately result in the observed developmental changes of facial shape. Quantification of local shape requires a perspective shift from landmark displacement towards local deformation [6,7]. The deformation perspective considers local shape differences as changes in tissues surrounding landmarks when one shape is deformed to fit the shape of another object. This perspective is also consistent with the biological process generating local shape differences, where deformation of tissue in between landmarks results in landmark displacement, not vice versa [8]. Electronic supplementary material, appendix figure S1 and appendix text S1 provide examples with detailed illustration of how deformation perspective generates insights into the morphogenetic process leading to the observed shape differences. Prevailing accounts of facial shape development have been largely based on the landmark displacement perspective. Adopting a deformation perspective enables deeper insights by unravelling how local deformations are spatio-temporally patterned in shaping facial developmental processes.

Facial growth and development have been equated to changes in the averages across age groups. However, mean, rate and variance of facial features provide complementary information that collectively portrays a comprehensive profile of growth dynamics. A multivariate quantification of shape variation allows for reconstruction of facial features which are developmentally constrained or diversified [9]. This information elucidates how developmental processes constrain or facilitate normal human facial variation.

The present study aimed to characterize facial growth and development with respect to mean, rate and variance of facial shape. This expanded scope, combined with a shift towards a deformation perspective, hopefully provides transformative insights into developmental patterns of facial regions with implications for clinical practice.

## 2. Material and methods

### 2.1. Study sample

This study is based on data from the 3D Facial Norms (3DFN) project [10]. The data repository contains three-dimensional facial images and demographic information on 2454 individuals of European ancestry 3 to 40 years of age. Participants of the 3DFN database were recruited in four US cities: Pittsburgh, Seattle, Houston and Iowa City. Inclusion criteria for participants are: free from conditions, trauma or surgical operations involving facial structures. Two participants were excluded in the study due to poor quality of three-dimensional facial images; 22 were excluded due to incomplete information on age, sex, height or weight. A total of 318 individuals with extreme values of height, weight or body mass index (BMI) were also excluded (electronic supplementary material, appendix text S2).

### 2.2. Facial phenotyping

Three-dimensional facial images of participants in the 3DFN project were obtained using the 3dMDface stereophotogrammetric optical system (3dMD, Atlanta, GA, USA). A template mesh comprising 7160 pseudolandmarks (Points placed automatically without regard to manually placed landmarks) were warped to each three-dimensional facial image using the MeshMonk toolbox in MATLAB [11]. As a result, 7160 homologous pseudolandmarks were obtained for each individual. The MeshMonk toolbox has been verified to be highly accurate (up to 1.26 mm) and reliable (inter-observer landmark digitization error of 0.40 mm) and is commonly used for digitizing landmarks in facial shape analysis [11]. Centroid size (CS) was calculated to reflect the overall size of each configuration [2].

Original landmark configurations of all individuals were jointly submitted to generalized Procrustes analysis to remove variation in location, size and orientation [12]. Symmetrization was performed to avoid confounding of facial asymmetry with further analyses [13]. Principal component analysis (PCA) of symmetrized Procrustes coordinates suggested that the first 98 eigenvectors collectively explained 99.0% of variance of the symmetric component of facial shape. Participants' principal component (PC) scores along the first 98 eigenvectors formed the basis of shape analysis. The analysis was performed using the Morpho package (v. 2.1.1) in R 4.3.1 [14].

### 2.3. Facial growth and development trajectories

Facial growth and development trajectories were established following a method established by Matthews *et al*. [3]. Weighted partial least-squares regression (wPLS) was applied to obtain predicted response variables and corresponding regression coefficients separately for each sex. For facial growth trajectories, the response variable was centroid size. For shape development trajectory, the response variables were PC scores along the 98 eigenvectors. The regression coefficients represent the rate of change in size or shape. The rate of shape development is a multi-dimensional vector that requires quantification by its magnitude and direction in PC space. The rate vector was analysed and visualized in the multi-dimensional space by incorporating all landmarks jointly, as opposed to calculating per-landmark rate as in Matthews *et al*. [3]. The weight of the *i*th individual evaluated at age *x_m_* was determined from a Gaussian kernel $w_{i}=(1/\surd 2\pi )e^{-({\left(x_{i}-x_{m}\right)}^{2}/2\sigma^{2})}$, where *σ* controls the bandwidth of the Gaussian kernel. This ensured that individuals aged closer to the evaluation age had a stronger influence on estimates. A bandwidth of 2.25 and 3 was used respectively for growth and development trajectory. Prediction of centroid size was restricted to 4 to 38 years of age and shape prediction was restricted to 5 to 37 years of age. Details regarding the determination of bandwidth and age range selected for prediction analysis are available in electronic supplementary material, appendix text S3.

The magnitude of sexual dimorphism of predicted facial shape during 5 to 37 years of age was determined by Procrustes distance and its statistical significance evaluated through 10 000 rounds of permutation of sex of the individuals. Generalized additive model (GAM) with penalized thin plate regression splines was applied to investigate nonlinear associations between Procrustes distance and age.

### 2.4. Developmental changes of facial shape variation

The magnitude of shape variation can be quantified through Procrustes variance, which is the sum of squared distance of individuals from the group mean divided by the group size. Established tool (the morphol.disparity function in the geomorph package in R) only allows for estimation of Procrustes variance when shape is linearly modelled. A custom routine gen.morphol.disparity was developed to allow for estimation of Procrustes variance for nonlinearly modelled shape. Details are provided in electronic supplementary material, appendix text S4. Estimation of Procrustes variance was performed for age groups in bins of 1 year from age 5 to 37 years. Pairwise comparisons were performed, and statistical significance was evaluated through 10 000 permutations. Sex-specific and pooled developmental trends of Procrustes variance were modelled through GAM.

To further explore specific facial features that contribute to changes of Procrustes variance, covariance structures of facial features were examined. Relative PCA was performed to identify shape features with high and low contrasts of variance between 6 to 9 years of age (females: 57; males 60) and 18 to 20 years of age (females: 181; males: 82) by sex [15]. The first five PCs explaining 70.9% of shape variance among individuals 6 to 20 years of age were retained for relative PCA, ensuring the sample size in both age groups was greater than 10 times the number of PCs retained [9]. Proportionality of covariance matrix of shape features at 6 to 9 years of age and 18 to 20 years of age, in addition to statistical significance regarding differences in successive relative eigenvalues, was examined using the likelihood ratio test. False discovery rate (FDR) adjustment was made to *p* values from tests of successive relative eigenvalues. Analysis was performed using the vcvComp package (v. 1.0.2) in R [9]. Patterns of facial features identified from relative PCA were reconstructed for visualization.

## 3. Results

2112 participants (females: 1290; males: 822) 3 to 40 years of age from the 3DFN project were included in the present study. Age distribution of the study participants is described in electronic supplementary material, appendix figure S2.

### 3.1. Growth changes of facial CS

**Figure 1.**
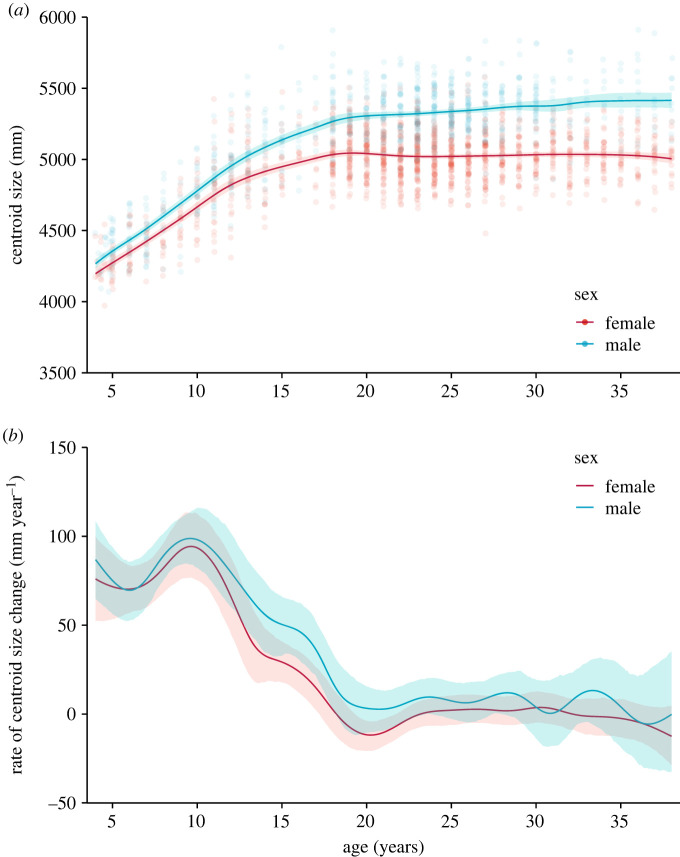
Age trajectories of facial CS. (*a*) Age trajectories of facial CS stratified by sex. (*b*) Trajectories of the instantaneous rate of facial CS changes stratified by sex. Shadings around the lines represent 95% confidence bands of the estimates.

Nonlinear pattern of change in facial CS was identified during 4 to 38 years of age (figure 1*a*). Facial CS was higher in males across all age groups and the gap between sexes widened with age. The highest growth rate in facial CS was at 10 years of age for both sexes (figure 1*b*), followed by a constant decline until 18 years of age. After 18 years of age, facial CS plateaued in both sexes. Larger sex differences in growth rate were observed at 14 to 18 years of age compared with earlier age periods.

### 3.2. Developmental changes of facial shape

**Figure 2.**
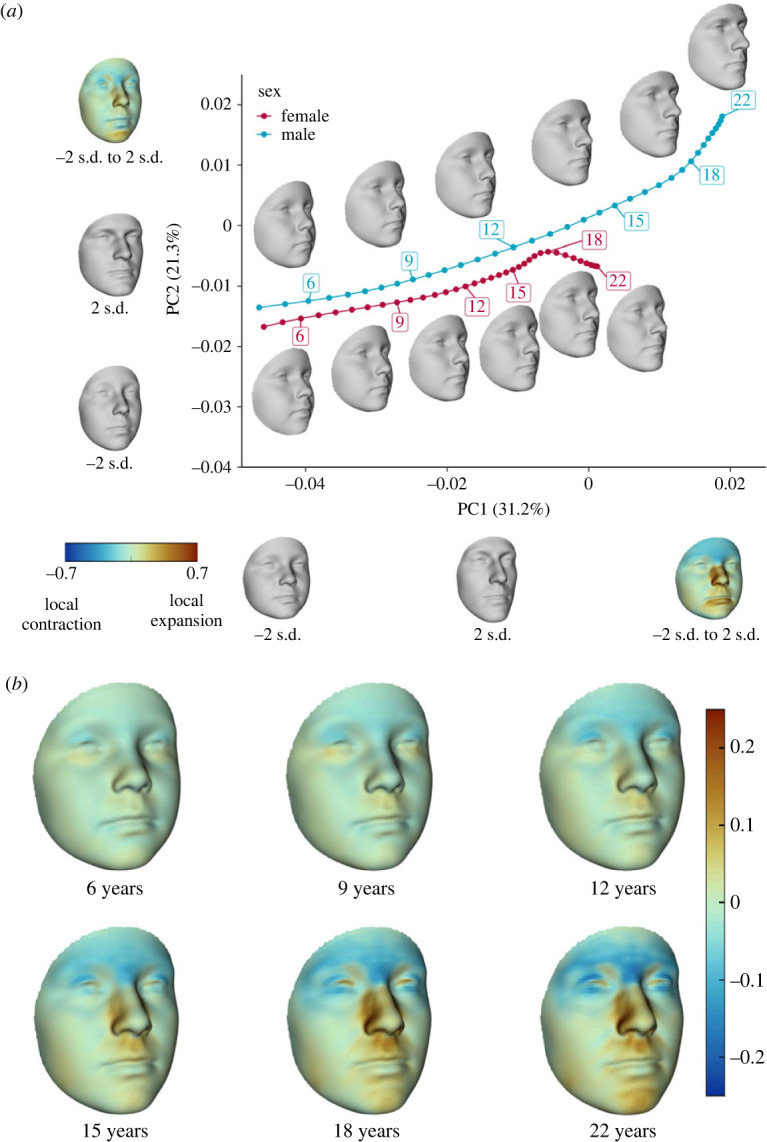
Age-related changes and sexual dimorphism of facial shape. (*a*) Age trajectories of female and male facial shape projected onto the first two PCs. Facial meshes along the trajectories illustrate the predicted facial shapes at key developmental stages. Numbers in parentheses represent the percentage of shape variance accounted for by the respective PC. Grey facial meshes along the PC axes illustrate predicted facial shapes 2 s.d. below and above the mean shape along the respective PC. Colour facial meshes illustrate the deformation from 2 s.d. below to 2 s.d. above the mean shape along the respective PC. The colour coding represents the percentage changes of the surface area of the meshes' triangular facets. Warm colour denotes expansion and cold colour denotes contraction. (*b*) Sexual dimorphism of predicted facial shape at key developmental stages. The colours represent deformation from predicted female to predicted male facial shape.

Facial shape was predicted from the wPLS model based on age intervals of 0.5 years from 5 to 37 years of age. Predicted facial shape was projected to the first two PCs of the study sample to establish facial shape development trajectories. Results from electronic supplementary material, appendix figure S3 suggest that changes in facial shape after 22 years of age become irregular and occupy only a small portion of the PC space. Based on these results, interpretation was restricted to changes prior to 22 years of age. Trajectories of facial shape development for both sexes were generally aligned with PC1, accounting for 31.2% of shape variance in the sample (figure 2*a*). PC1 was characterized by facial elongation and forward displacement of the nasolabial region. Similar shape features were identified from superimposition of facial meshes at 6 and 18 years of age (electronic supplementary material, appendix figure S4A,B). Heatmaps of local deformation suggested a relative contraction along the forehead and relative expansion within nasal lateral walls and the lower labial region. Sex is differentiated along PC2 and is characterized by contraction in the nasal root and expansion in the nasal lateral walls, nasal alar and chin. These local deformations resulted in forward displacement of the frontonasal and chin region and backward displaced cheek.

**Figure 3.**
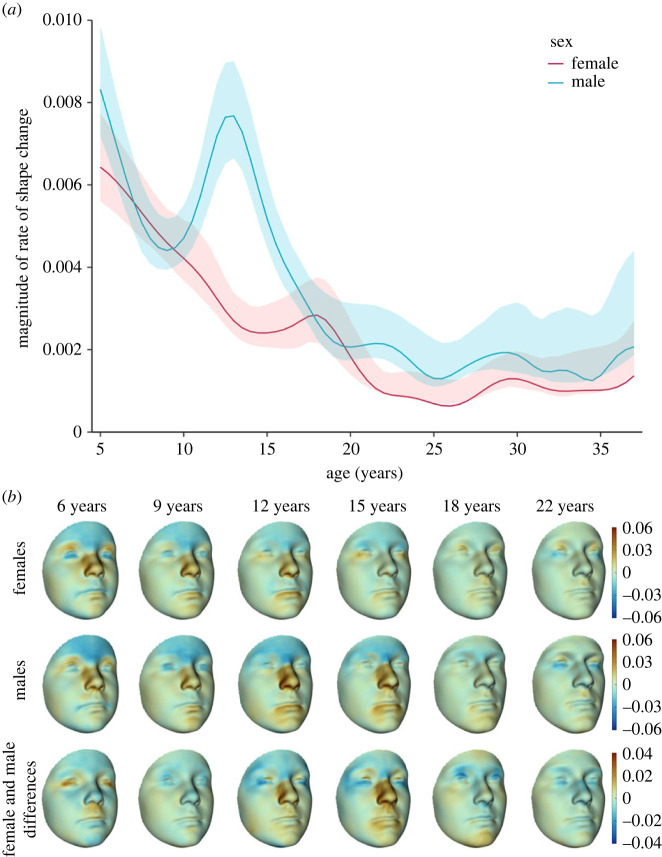
Rate of changes of facial shape and its sexual dimorphism. (*a*) Trajectories of magnitude of instantaneous change rate of facial shape stratified by sex. (*b*) Morphological effect of the instantaneous change rate of facial shape at key developmental stages. Upper row: Deformation from predicted female facial shape at respective age to the target shape (the sum of the predicted shape and the instantaneous rate at the same age). Middle row: Deformation from predicted male facial shape at respective age to the target shape (the sum of the predicted shape and the instantaneous rate at the same age). Lower row: Sexual dimorphism of the morphological effect of the instantaneous rate of changes of facial shape. This is evaluated as the deformation from the reference shape (the sum of predicted female facial shape and the instantaneous rate for females at the same age) to the target shape (the sum of predicted female facial shape and the instantaneous rate for males at the same age). Results are presented using predicted female faces to construct the reference and target shape, but highly similar patterns are obtained when predicted male faces are used instead.

Sexual dimorphism in facial shape emerged at 6 years of age (electronic supplementary material, appendix table S1) and persisted throughout subsequent ages with differences increasing significantly (*p* < 0.001) and nonlinearly (electronic supplementary material, appendix figure S5). Rate of facial shape development declined with age monotonically in females (figure 3*a*). In males, however, a markedly heightened rate of development was observed during 12 to 15 years of age. This corresponded to the large shape development along PC1 during this period (figure 2), which was primarily driven by shape development in the nasal region (figure 3*b*). In both sexes, a perceptible relative contraction in the forehead region was identified at 6 to 15 years of age, with the effect stronger in males (figure 3*b*). During 6 to 9 years of age, expansion in the supraorbital region was also observed in both sexes, which is relatively weaker for males at age 9 years. At 12 to 15 years of age, expansion was confined to the medial supraorbital region and was stronger in males. The nasal region underwent continuous shape change during 6 to 15 years of ages, characterized by local expansion in structures surrounding the nasal dorsum, including root, lateral walls and nasal alar. The local expansion was stronger in females during 6 to 9 years of age and in contrast, was stronger in males 12 to 15 years of age. Sex difference in nasal shape development during 12 to 15 years of age is one of the most conspicuous sexually dimorphic patterns of facial shape development, resulting in noticeable sex difference in nasal shape at 18 and 22 years of age (figure 2*b* and electronic supplementary material, appendix figure S4C). Relatively miniscule local deformation was identified in the cheek region with age. Sex difference in the development of upper and lower labial regions was marked by comparatively stronger local expansion in males as compared with females at 15 and 18 years of age, respectively.

### 3.3. Developmental changes of variation of facial shape

**Figure 4.**
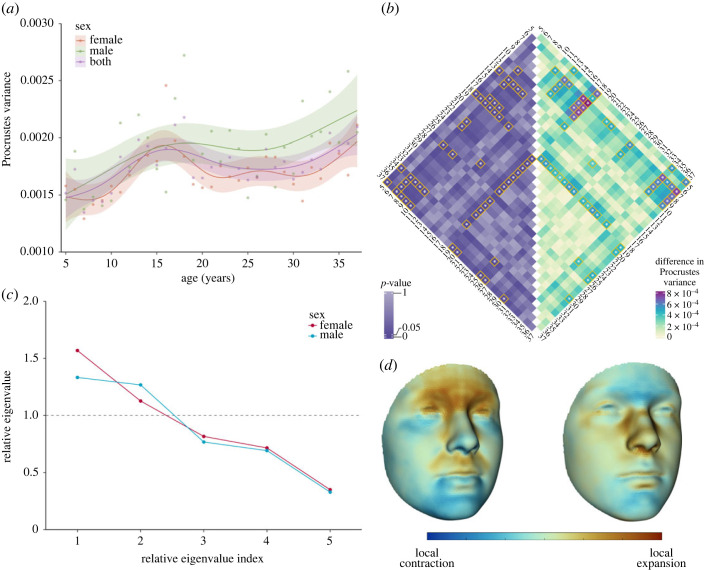
Age-related changes of variation of facial shape. (*a*) Age trajectories of Procrustes variance of facial shape by sex. The nonlinear relationship was modelled using a generalized additive model. Shadings around the lines represent 95% confidence bands of the estimates. (*b*) Pairwise differences in Procrustes variance between age groups from 5 to 37 years of age. The absolute values of the differences are displayed in the right triangle and corresponding *p* values are displayed in the left triangle. Significant differences are indicated by asterisks and are bordered in yellow. (*c*) Relative eigenvalues of the covariance matrix of shape variables for individuals 6 to 9 years of age relative to that of individuals 18 to 20 years of age. (*d*) Facial shape features most developmentally diversified in females (left coloured mesh) and males (right coloured mesh).

GAM suggested that Procrustes variance increased nonlinearly with age in both sex-specific (females: *p* = 0.002; males: *p* = 0.023) and pooled (*p* < 0.001) analysis (figure 4*a*). Pairwise comparisons of Procrustes variance across age groups suggested significant differences between early and late stages of facial shape development (figure 4*b*).

Test of proportionality (*p* = 0.036) suggested that covariance structure of female facial shape at 6 to 9 years of age differed from 18 to 20 years of age by more than merely difference in scale, suggesting shape features that varied among 6 to 9 years of age differed from features that varied among 18 to 20 years of age. Relative PCA (figure 4*c*) of the two groups revealed generalized variance of facial shape in 6 to 9 years of age was 36.5% of the variance in 18- to 20-year-olds, consistent with increased shape variation with development. In males, generalized variance of shape features at 6 to 9 years of age is 29.8% of variance in 18 to 20 years of age. Test of proportionality failed to identify difference in covariance structure between the two groups beyond simple difference by scaling factor (*p* = 0.141). However, tests of successive relative eigenvectors in both sexes revealed significant differences in relative eigenvalues of the fourth and fifth relative eigenvector (Female *p*_FDR_ = 0.031; Male *p*_FDR_ = 0.042), highly suggesting the need to investigate the fifth relative eigenvector individually.

The fifth relative eigenvector represented shape features with the largest amount of excessive variance in 18 to 20 years of age compared with 6 to 9 years of ages. Shape features in females are characterized by contrasting local deformations in the frontal, lateral nasal and upper labial regions versus lower labial and chin regions (figure 4*d*) resulting in contrasting displacement of the frontonasal and labial regions relative to other facial features (electronic supplementary material, appendix figure S6A,B). In males, contrasting local deformation was observed in the frontal and chin regions compared with the nasal lateral walls, nasal alar and columella regions, resulting in contrasting relative displacement of frontal and chin regions compared with other facial features (electronic supplementary material, appendix figure S6C,D).

Concordance of spatial distribution of local deformations captured by the fifth relative eigenvector (figure 4*d*) with local deformations reflecting rate of facial shape development at key developmental stages from 6 to 22 years of age (figure 3*b*) was examined using the Pearson correlation coefficient. Moderate-to-high correlation with growth rates at 9, 12 and 15 years of age was observed in both sexes (electronic supplementary material, appendix table S2).

## 4. Discussion

Our study identified key developmental stages for growth and development of facial size and shape. Transformative insights into developmental patterns of facial regions were obtained by investigating the spatio-temporal dynamics of local deformations resulting in observed morphological changes. Overall, variance of facial shape increased nonlinearly with age. Facial features with accelerated growth rates tended to exhibit greater degree of among-individual variability with development.

Female facial size has been suggested to plateau at 14 years of age [3]. However, previous predictions of facial size were truncated at 16 years old. By modelling a more extensive age period, our study suggests continuation of growth in facial size until 18 years of age in both sexes. The traditional viewpoint that facial size growth plateaus in females at 14 years of age but continues in males may result from an accentuated sex difference in growth rate after 14 years of age.

Our study suggests sexual dimorphism in facial shape emerges at 6 years of age. The literature regarding sex difference in facial shape prior to 5 years of age is inconclusive [3,16]. Our findings reveal that sexual dimorphism increases nonlinearly with age and supports Enlow and Hans’ hypothesis that sexually dimorphic facial features become apparent during female pubertal growth spurt [17]. Similar to our findings, geometric morphometric analysis of 32 cephalograms from the Bolton–Brush Growth Study suggested a peak of male facial shape development at 15 years of age [18]. In addition, our study further suggests that the rapid shape development at 15 years of age is attributable to the frontal, nasal and labial regions. Both studies were unable to identify an unambiguous peak in females. Our findings are in agreement with established accounts of facial sexual dimorphism where females have less protrusive nose, more prominent cheeks and a gracile lower face [19].

A series of transformative insights on patterns of facial shape development were obtained using the deformation perspective. Developmental change in nasal shape is one of the most conspicuous features in both sexes. The visually apparent displacement of nasal bridge is generated from local expansion in surrounding tissues whereas shape changes of the nasal bridge are less pronounced. A previous report of a developmentally more sloping forehead [3] was found to be due to expansion in the supraorbital region. This local expansion is a result of developmental divergence of the inner and outer table of the frontal bone, creating space for the frontal sinus [17]. The more pronounced expansion of the medial supraorbital region in males identified in this study results in a smooth transition from the forehead to the markedly protrusive male nose and relatively deeper positioned eyes in males. Observations of a pervasive contraction in the forehead, although a major developmental feature, cannot be inferred from studies using a landmark displacement perspective. The relative contraction results from continued growth of the mid- and lower face to accommodate increased respiratory requirements and masticatory function [17].

Local expansion of the lower labial region is consistently observed during 6 to 18 years of age, resulting in the developmentally protrusive lower lip. The local expansion in upper lip observed in males 15 years of age is not found in females, and as a result, females have less protrusive upper lip. We also found that the cheek region is developmentally less prominent, which may result from the protrusive growth of the forehead, nose and lips, and probably not due to local shape changes of the cheek. Female cheeks present as puffy due to the less protrusive growth of surrounding structures. Sites of local deformation identified in this study are suggestive of potential facial growth factors which may aid in designing plans for orthodontic treatment and maxillofacial and plastic surgeries. For example, the effective harnessing of growth patterns can enable shorter treatment duration and less invasive procedures to ensure desirable and stable outcomes.

Developmental changes within variational properties of facial shape are rarely investigated despite its potential in unravelling the role of developmental processes for shaping facial variations. We found that variation in facial shape increased with development, and in addition, the nature of shape variation also changes with development. The relatively small sample size for males may have limited the statistical power of the test of proportionality, which might explain the lack of deviation from the null hypothesis of proportionality in covariance matrices between 6 to 9 and 18 to 20 years of age. Nevertheless, significant difference in relative eigenvalue between the fourth and fifth relative eigenvector was consistently observed in both sexes. Facial features with the strongest pattern of developmental diversification are aligned with features showing the fastest rate of shape development. Developmental process is therefore in itself a potential catalyst of morphological variation in Europeans.

This study is one of the largest to date investigating facial growth and development. The constraints of our study limited the number of individuals below 18 years of age but is comparable with the previous large studies. We mitigated the effect of proportionally smaller number of younger individuals using the Gaussian kernel weighting scheme during trajectory modelling. Nevertheless, a larger sample size for younger individuals would allow for more robust results from relative PCA.

## 5. Conclusion

Our study moves beyond traditional descriptive illustration towards an explanatory insight into patterns of facial growth and development through a deformation perspective. Using these findings can assist in establishing facial growth charts which encompass more broad measures than mean shape alone. This study also provides a methodological framework with publicly available custom routines which are useful for morphometric investigation of face or other morphometric targets where growth and development are of interest.

## Data Availability

All three three-dimensional facial images are available through the FaceBase Consortium controlled-access repository. Template facial mesh and MeshMonk toolbox for landmark digitization are available: https://gitlab.kuleuven.be/mirc/meshmonk. Scripts to establish facial growth and development trajectories through the weighted partial least-squares regression model are available: https://github.com/harrymatthews50/Modelling_Craniofacial_Growth_Trajectories. The custom routine for estimation of Procrustes variance from nonlinearly modelled shape data is publicly available: https://github.com/Patrick-Wen/gen.morphol.disparity and has been archived within the Zenodo repository: https://zenodo.org/records/10311951 [20]. Example script to run this custom function is provided in electronic supplementary material, appendix text S4. All other analyses are based on publicly available packages in R software as described in the main text. Supplementary material is available online [21].
